# Relative validation of the KiGGS Food Frequency Questionnaire among adolescents in Germany

**DOI:** 10.1186/1475-2891-10-133

**Published:** 2011-12-07

**Authors:** Julia Truthmann, Gert BM Mensink, Almut Richter

**Affiliations:** 1 Technische Universität München, School of Management, Marketing and Consumer Research, Alte Akademie 16, D-85350 Freising, Germany; 2 Robert Koch Institute, Department of Epidemiology and Health Reporting, Post box 65 02 61, D-13302 Berlin, Germany

**Keywords:** adolescents, dietary surveys, nutrition assessment, Germany, population characteristics, epidemiology

## Abstract

**Background:**

The aim of this study was to determine the relative validity of the self-administered Food Frequency Questionnaire (FFQ) "What do you eat?", which was used in the German National Health Interview and Examination Survey for Children and Adolescents (KiGGS 2003-2006).

**Methods:**

The validation was conducted in the EsKiMo Nutrition Module, a subsample of KiGGS. The study population included 1,213 adolescents aged between 12 and 17. A modified diet history interview DISHES (Dietary Interview Software for Health Examination Studies) was used as the reference method. In order to compare the food groups, the data assessed with both instruments were aggregated to 40 similar food groups. The statistical analysis included calculating and comparing Spearman's correlation coefficients, calculating the mean difference between both methods, and ranking participants (quartiles) according to food group consumption, including weighted kappa coefficients. Correlations were also evaluated for relative body weight and socioeconomic status subgroups.

**Results:**

In the total study population the Spearman correlation coefficients ranged from 0.22 for pasta/rice to 0.69 for margarine; most values were 0.50 and higher. The mean difference ranged between 1.4% for milk and 100.3% for pasta/rice. The 2.5 percentiles and 97.5 percentiles indicated a wide range of differences. Classifications in the same and adjacent quartile varied between 70.1% for pasta/rice and 90.8% for coffee. For most groups, Cohen's weighted kappa showed values between 0.21 and 0.60. Only for white bread and pasta/rice were values less than 0.20. Most of the 40 food groups showed acceptable to good correlations in all investigated subgroups concerning age, sex, body weight and socio-economic status.

**Conclusions:**

The KiGGS FFQ showed fair to moderate ranking validity except for pasta/rice and white bread. However, the ability to assess absolute intakes is limited. The correlation coefficients for most food items were similar for normal weight and overweight as well as for different socio-economic status groups. Overall, the results of the relative validity were comparable to FFQs from the current literature.

## Background

Diet plays an important role for physical development and health status in the early life stages. Behavioural aspects contributing to disease risk in adulthood often originate in childhood and adolescence [1]. The accurate assessment of dietary intake is essential in order to investigate the relationship between diet and health [2]. Large studies and accurate methods are necessary for many nutrition research questions, but these are expensive and time consuming. Food Frequency Questionnaires (FFQs) assess the usual diet of study participants by asking the respondents about the frequency and portion size of predefined foods. In general, FFQs are time and cost efficient and have therefore become established in estimating usual food intake in population studies [3]. However, FFQs are known to have limitations and to be prone to measurement errors [4]. Especially children and adolescents have problems estimating the usual portion sizes and remembering their diet over a long time period. The reasons are, among others, unstructured eating patterns and more frequent meals outside the home [5]. Although some FFQ validation studies have been conducted for adolescents [6-10], validity in subgroups concerning age, sex, body weight, socio-economic status, etc. was not well examined [11]. Since biased results, even after stratification, can lead to wrong associations, the relative validity of FFQs should be determined by comparison with an established method in the population of interest.

A self-administered, semi-quantitative FFQ was used in the German National Health Interview and Examination Survey for Children and Adolescents (KiGGS 2003-2006) [12]. The main purpose of this questionnaire was to rank participants according to their food intake, but not to estimate the complete diet. Data are used to analyse diet-disease associations, as confounding variables within other exposure-disease associations, and to compare consumption patterns within population groups. In the 2006 EsKiMo study (Eating Study as a KiGGS Module), the detailed food consumption of adolescents was assessed by means of a modified diet history interview (DISHES) in a subsample of KiGGS participants. Furthermore, the participants were asked to complete the KiGGS FFQ a second time. Although EsKiMo was not primarily designed as a validation study, it enabled a food-group validation of the KiGGS FFQ using DISHES, a more comprehensive dietary assessment method, which was already validated for adults [13]. Since the EsKiMo module included a large representative sample of German adolescents, validity in subgroups concerning age, sex, body weight, and socio-economic status was also verified.

## Methods

### Study design

The KiGGS study was conducted between 2003 and 2006 by the Robert Koch Institute. It collected comprehensive, nationally representative data on the health of children and adolescents [14]. The aim of this nationwide survey was to give an overview of many relevant health aspects among children and adolescents. The survey included 17,641 participants aged 0 to 17 who lived in Germany and were registered in local population registries. Children and adolescents with a migration background were also included [15]. Special efforts were undertaken to include migrants: e.g. oversampling and translation of letters of invitation, information material and health questionnaires. The study consisted of the KiGGS core survey and five additional modules: the Iodine Module, the Nutrition Module (EsKiMo), the Mental Health Module (BELLA), the State Module "Schleswig-Holstein", the Motor Activity Module (MoMo), and the Environmental Module (KUS), which aimed to explore certain health-relevant topics in more detail. It would have been too costly and time intensive to conduct all the measurements in the total sample and would probably have reduced the compliance and response rate. In the core survey participants were enrolled in two steps. First, 167 sample points were chosen randomly, but in proportion to the size of the respective federal state and community. Within these points, persons were randomly selected, stratified by age, from local population registries. All participants were interviewed and investigated comprehensively about their health history and status, health behaviour, socio-demographic characteristics, etc. The FFQ "What do you eat?" was used to assess the usual diet. This questionnaire exists in two versions which differ only in the form of address. One was to be completed by the parents of the 1- to 10-year-olds, with questions formulated as "How often did your child eat...?". The other questionnaire was to be completed by the participants aged 11 to 17, with questions formulated as "How often did you eat...?".

The EsKiMo study was conducted from January to December 2006. The participants in EsKiMo were randomly selected from the KiGGS sample and stratified by age and sample point. The rationale was that about one hundred boys and girls were chosen per age group for statistically sound analyses. The validation was conducted with dietary data from 1,272 adolescents aged 12 to 17 years. The FFQ was sent to the EsKiMo participants by post three to four weeks prior to a local visit for the more comprehensive and detailed diet history interview (DISHES interview). Both instruments therefore cover largely the same time frame. The seasonality of diet was reflected at the group level by the equal distribution of the assessment over the year. Food consumption data from both methods were converted to mean intakes as grams per day, and food groups from the DISHES interview were aggregated to food groups comparable to those of the FFQ. The survey was approved by the German Federal Data-Protection Office and by the Ethics Committee of Charité University Medicine (university hospital). Respondents were informed in detail about the study objectives, interview and examination procedures, as well as the handling of data records and analysis under pseudonymous conditions, and gave their written consent. Design and methods are described in detail elsewhere [14,16].

### Dietary assessment

The self-administered FFQ "What do you eat" was developed at the Robert Koch Institute to assess the usual intake of several food groups in the KiGGS core survey (2003-2006). The food groups most often consumed by children and adolescents were selected based on data from previous surveys and the advice of nutrition survey experts [17]. Questions on the frequency and the amount of 45 food items consumed "during the last few weeks" were included. Additional questions related to specific nutritional demands (multivitamin tablets, convenience foods, light products). The frequency of consumption was assessed using ten response categories: never, once a month, two to three times a month, once or twice a week, three to four times a week, five to six times a week, once a day, two to three times a day, four to five times a day, more than five times a day. In addition, participants were asked to indicate the portion size of the food items, which was given in five item-specific categories. Several pictures were used to illustrate portion sizes. The time frame "during the last few weeks" for the FFQ was based on pre-test experience, since some participants reported that it was difficult to give an answer for exactly "the last four weeks". However, the predefined answer categories for the frequency of consumption imply a time frame of about four weeks, since the lowest frequencies relate to a frequency per month (once a month, two to three times a month). The FFQ and a covering letter were sent to the respondents by postal mail three to four weeks prior to the visit. The first page of the FFQ provides instructions on completing the questionnaire. During the survey period a telephone hotline offered support with completing the questionnaire. Furthermore, support was offered when questionnaires were collected on local visit for the DISHES interview. The development process and design are described in detail elsewhere [17].

The DISHES interview is a modified diet history interview for assessing the usual dietary intake, with a reference period of the last four weeks. This was used as reference instrument. The DISHES software facilitates a standardized, structured and interviewer-guided assessment. The procedure has a meal-based structure similar to many 24-hour recall instruments. It is standardized, but still open-ended and allows the assessment of all possible food items in detail. The DISHES interview was conducted by trained nutritionists at the residence of the participants. First, usual meal patterns were obtained. In the next step, food intakes consumed during each meal were assessed by a check list. Subsequently, the frequency and portion size of each food consumed at the different meals was determined in detail. Additional food items could be chosen by searching the food code database. In general, estimation of portion sizes was facilitated using standardized tableware models. In addition, a picture book adapted from the EPIC-SOFT Picture Book [18] could be used to determine the portion size of selected food items. The DISHES software codes food items and connects the codes with the German Food Code and Nutrient Database (BLS II.3), which includes 10,654 food codes [19]. For the EsKiMo study, the software was adapted for the target group of adolescents (DISHES Junior). Additional foods (1,225 food codes), not yet available in the BLS but often consumed by adolescents, were incorporated into the database. The average duration of an interview in the EsKiMo Study was 49 minutes. The instrument had been previously validated for adults [13] and used in several national nutrition surveys [16,20,21].

In the KiGGS study (2003-2006), the parents were asked about their income, occupational status and education. This information was used to calculate a family socio-economic status index, developed for the survey. The index was categorized into low (3-8 points), medium (9-14 points) and high (15-21 points) [22]. According to this index, 27.5% of the KiGGS participants were allocated to the low, 45.4% to the medium, and 27.1% to the high socio-economic status group [23]. Furthermore, the body weight and height of the adolescents was assessed by standardized measurement. The body mass index (BMI) was calculated from body height and weight. According to the Kromeyer-Hauschild method, participants with a BMI above the 90^th ^percentile of the age- and gender-specific reference values were categorized as overweight [24].

### Data and statistical analysis

The 45 FFQ items were aggregated to 40 food groups to enable a direct comparison of the two instruments. The FFQ items "fresh fruits" and "tinned fruits" were aggregated to fruits, and "cooked", "frozen", "tinned" and "raw vegetables" were aggregated to vegetables, since the original differentiation is not provided within the DISHES data. Furthermore, chocolate was added to the sweets group. Food frequency data were recoded into times of servings per month (one month being defined as 28 days). The arithmetic mean was used for frequency bands, and the frequency "more than five times a day" was set to six times a day. Portion categories were converted into gram amounts using predefined standard portion sizes. The average food-group intake was calculated by multiplying the frequency and portion size. For further information on the recoding of frequency and portion-size data, see Additional file 1. Food-level data were converted using SAS version 9.2 (SAS Institute, Cary, NC, USA). For most food groups, the food consumption was not normally distributed. Nonparametric Spearman rank-correlation coefficients were therefore calculated. Correlation coefficients were calculated for all participants and stratified by sex, age group, BMI, and socio-economic status. The commonly desired outcome from an FFQ is a good-ranking capability of participants [25]. To evaluate the agreement in ranking, participants were grouped into quartiles for each food group. Construction of quartiles was impossible for food groups where more than 25% of participants reported no consumption. Zero consumers were therefore defined as one group and the remaining participants grouped into tertiles. This was necessary for the following 20 food groups (percentages of zero consumers FFQ;DISHES): sport/energy drinks (64%;92%), tap water (46%;82%), fruit/herbal tea (33%;58%), black/green tea (73%;88%), coffee (69%;74%), breakfast cereals (17%;33%), brown bread (11%;39%), butter (31%;41%), margarine (46%;42%), cream cheese (37%;59%), eggs (13%;26%), fish (23%;33%), pasta/rice (0%;28%), cookies (17%;35%), ice cream (12%;30%), cream desserts/pudding/rice pudding (29%;41%), pancakes (29%;55%), sweet spreads (23%;37%), hazelnut spread (28%;44%), and nuts (52%;70%). Classification into the same, adjacent and opposite quartile or group was subsequently calculated. In addition, the degree of agreement was evaluated with the weighted kappa coefficient (κ_w_) using the formula [26]:

$$\kappa_{\text{w}}=\frac{{\text{O}}_{\text{w}}-{\text{C}}_{\text{w}}}{1-{\text{C}}_{\text{w}}}$$

For this, a cross table (4 × 4) of frequencies was calculated for each food group. The observed proportion of agreement (O_w_) and the expected proportion of agreement by chance (C_w_) were derived. The weighting factors were 1 for complete agreement (same quartile), 0.66 for persons differing in one category (adjacent quartile), 0.33 for persons differing in two categories, and 0 for complete disagreement (opposite quartile). The mean intakes derived from the FFQ and the mean differences between both methods were calculated according to the formula: Mean of difference = Mean (FFQ - DISHES). Furthermore, the mean % of difference was calculated according to the formula:

$$\text{Difference}\;\left(\%\right)=\frac{\text{Mean}\;\left(\text{FFQ}-\text{DISHES}\right)}{\left(\text{Mea}{\text{n}}_{\text{FFQ}}+\text{Mea}{\text{n}}_{\text{DISHES}}\right)∕2}*100$$

The 2.5 percentiles and the 97.5 percentiles of the difference were calculated. This represents the range of 95% of all differences. All statistical analyses were performed using SPSS version 18.0 (SPSS Inc., Chicago, Illinois, USA). Non-overlapping 95% confidence intervals were considered statistical significant.

## Results

The present analysis included 1,249 EsKiMo participants, who completed both instruments (FFQ and DISHES interview). Within the core KiGGS study, participants were excluded from the analysis of FFQ data if they reported having consumed over six litres of beverages and over four kilos solid food, or if there were more than 20 food items missing. Since the validation study is primarily for the evaluation of KiGGS, we used the same criteria. Thirty respondents had too many missing values for frequency questions and were excluded from the validation. Three respondents were excluded because of implausibly high consumption data. Finally, the sample for the statistical analysis included 1,213 adolescents aged 12 to 17. The characteristics of the validation sample are shown in Table 1. The sample includes 582 boys and 631 girls.

**Table 1:** Characteristics of study participants

	n	%
Sex		
Male	582	48.0
Female	631	52.0
Age group		
12-13 years	416	34.3
14-15 years	427	35.2
16-17 years	370	30.5
Body weight (BMI^a^)		
Under-/normal weight (≤ P90)	1005	82.9
Overweight (>P97)	201	16.6
Missing	7	0.6
Socio-economic status^b^		
Low	249	20.5
Medium	617	50.9
High	331	27.3
Missing	16	1.3

Abbreviation: BMI (body mass index), P (Percentile)

^a ^According to Kromeyer-Hauschild et al. [24]

^b ^According to Winkler [22]

### Correlations

Table 2 shows the correlation between the two methods in different food groups. The correlation coefficients for the total group of participants varied between 0.22 (pasta/rice) and 0.69 (margarine); most values were 0.50 and higher. Correlation coefficients between 0.3 and 0.5 were observed for potato products, pancakes, meat, vegetables, cakes/pastries, tap water, cookies, poultry, nuts, bread and sport/energy drinks. Only for the food group pasta/rice was the correlation coefficient less than 0.3.

**Table 2:** Correlation coefficients (95% CI) of food group intake between both methods by sex and age*

Food group	all n = 1213	Sex		Age group		
		
		Male n = 582	Female n = 631	12-13 years n = 416	14-15 years n = 427	16-17 years n = 370
Margarine	0.69	0.65 (.60-.69)	0.73 (.69-.76)	0.71 (.66-.75)	0.67 (.61-.72)	0.69 (.63-.74)
Coffee	0.68	0.66 (.61-.70)	0.69 (.65-.73)	**0.57 (.50-.63)**	0.66 (.60-.71)	**0.70 (.64-.75)**
Hazelnut spread	0.67	0.63 (.58-.68)	0.71 (.67-.75)	0.68 (.62-.73)	0.68 (.63-.73)	0.64 (.58-.70)
Sweet spreads	0.66	0.65 (.60-.69)	0.67 (.62-.71)	0.65 (.59-.70)	0.64 (.58-.69)	0.69 (.63-.74)
Breakfast cereals	0.66	0.67 (.62-.71)	0.64 (.59-.68)	**0.58 (.51-.64)**	0.66 (.60-.71)	**0.71 (.66-.76)**
Milk	0.66	0.66 (.61-.70)	0.65 (.60-.69)	0.64 (.58-.69)	0.69 (.64-.74)	0.65 (.59-.71)
Mineral water	0.66	0.68 (.63-.72)	0.63 (.58-.67)	0.61 (.55-.67)	0.67 (.61-.72)	0.68 (.62-.73)
Soda	0.65	0.68 (.63-.72)	0.60 (.55-.65)	0.63 (.57-.68)	0.64 (.58-.69)	0.68 (.62-.73)
Butter	0.63	0.63 (.58-.68)	0.62 (.57-.67)	0.58 (.51-.64)	0.67 (.61-.72)	0.63 (.56-.69)
Fruits	0.63	0.62 (.57-.67)	0.62 (.57-.67)	0.60 (.53-.66)	0.63 (.57-.68)	0.66 (.60-.71)
Fruit/herbal tea	0.62	0.60 (.55-.65)	0.63 (.58-.67)	**0.67 (.61-.72)**	**0.54 (.47-.60)**	0.64 (.58-.70)
Green/black tea	0.62	0.58 (.52-.63)	0.65 (.60-.69)	**0.56 (.49-.62)**	**0.71 (.66-.75)**	**0.57 (.50-.64)**
Cheese	0.61	0.60 (.55-.65)	0.62 (.57-.67)	0.60 (.53-.66)	0.60 (.54-.66)	0.62 (.55-.68)
Fish	0.60	0.57 (.51-.62)	0.63 (.58-.67)	**0.56 (.49-.62)**	**0.55 (.48-.61)**	**0.70 (.64-.75)**
Ice cream	0.59	0.56 (.50-.61)	0.62 (.57-.67)	0.61 (.55-.67)	0.59 (.52-.65)	0.57 (.50-.64)
Eggs	0.58	0.54 (.48-.60)	0.60 (.55-.65)	0.61 (.55-.67)	0.56 (.49-.62)	0.57 (.50-.64)
Cream cheese	0.58	0.52 (.46-.58)	0.62 (.57-.67)	0.62 (.56-.68)	0.54 (.47-.60)	0.57 (.50-.64)
Salty snacks	0.57	0.58 (.52-.63)	0.55 (.49-.60)	0.60 (.53-.66)	0.56 (.49-.62)	0.55 (.47-.62)
Ketchup/mayonnaise	0.56	0.57 (.51-.62)	0.54 (.48-.59)	0.58 (.51-.64)	0.51 (.44-.58)	0.61 (.54-.67)
Curd^1^	0.56	0.54 (.48-.60)	0.57 (.51-.62)	0.52 (.45-.59)	0.58 (.51-.64)	0.58 (.51-.64)
Meat products	0.55	0.49 (.43-.55)	0.58 (.53-.63)	0.50 (.42-.57)	0.55 (.48-.61)	0.60 (.53-.66)
Potatoes	0.54	0.52 (.46-.58)	0.55 (.49-.60)	0.48 (.40-.55)	0.60 (.54-.66)	0.57 (.50-.64)
Juice	0.54	0.53 (.47-.59)	0.55 (.49-.60)	0.58 (.51-.64)	0.48 (.40-.55)	0.57 (.50-.64)
Fast food^2^	0.53	0.50 (.44-.56)	0.47 (.41-.53)	0.49 (.41-.56)	**0.45 (.37-.52)**	**0.62 (.55-.68)**
Soup	0.52	0.51 (.45-.57)	0.53 (.47-.58)	0.50 (.42-.57)	0.54 (.47-.60)	0.51 (.43-.58)
Sweets^3^	0.52	0.51 (.45-.57)	0.51 (.45-.57)	0.50 (.42-.57)	0.49 (.41-.56)	0.55 (.47-.62)
Pudding/rice pudding	0.50	0.50 (.44-.56)	0.49 (.43-.55)	0.46 (.38-.53)	0.51 (.44-.58)	0.53 (.45-.60)
Potato products	0.49	0.47 (.40-.53)	0.50 (.44-.56)	0.50 (.42-.57)	0.42 (.34-.50)	0.55 (.47-.62)
Pancakes	0.49	0.47 (.40-.53)	0.51 (.45-.57)	0.49 (.41-.56)	0.43 (.35-.50)	0.55 (.47-.62)
Meat	0.47	0.41 (.34-.48)	0.47 (.41-.53)	0.44 (.36-.51)	0.45 (.37-.52)	0.52 (.44-.59)
Vegetables	0.44	**0.58 (.52-.63)**	**0.40 (.33-.46)**	0.47 (.39-.54)	0.41 (.33-.49)	0.45 (.36-.53)
Cakes/pastries	0.44	0.44 (.37-.50)	0.43 (.36-.49)	0.39 (.31-.47)	0.47 (.39-.54)	0.46 (.38-.54)
Tap water	0.43	0.44 (.37-.50)	0.41 (.34-.47)	0.41 (.33-.49)	0.43 (.35-.50)	0.43 (.34-.51)
Cookies	0.41	0.44 (.37-.50)	0.40 (.33-.46)	0.43 (.35-.51)	0.44 (.36-.51)	0.36 (.27-.45)
Poultry	0.39	**0.30 (.22-.37)**	**0.46 (.40-.52)**	0.39 (.31-.47)	0.37 (.29-.45)	0.43 (.34-.51)
Nuts	0.38	0.37 (.30-.44)	0.38 (.31-.44)	0.46 (.38-.53)	0.40 (.32-.48)	0.38 (.29-.46)
Brown bread^4^	0.35	0.36 (.29-.43)	0.34 (.27-.41)	0.32 (.23-.40)	0.32 (.23-.40)	0.43 (.34-.51)
White bread^5^	0.31	0.23 (.15-.31)	0.33 (.26-.40)	0.27 (.18-.36)	0.31 (.22-.39)	0.62 (.55-.68)
Sport/energy drinks	0.31	**0.38 (.31-.45)**	**0.18 (.10-.25)**	**0.32 (.23-.40)**	**0.31 (.22-.39)**	**0.29 (.19-.38)**
Pasta/rice	0.22	0.19 (.11-.27)	0.26 (.19-.33)	0.17 (.08-.26)	0.25 (.16-.34)	0.25 (.15-.34)

Mean	0.53	0.52	0.53	0.52	0.52	0.56

Abbreviation: CI (confidence interval)

*Non-overlapping 95% confidence intervals (bold) were considered statistically significant

^1^Curd, yoghurt, soured milk

^2^Burger, doner kebab, fried/grilled sausage, curried sausage

^3^Sweets, fruit chews, chocolate

^4^Brown bread, brown bun

^5^White bread, white bun

### Subgroups

Correlation coefficients were similar for boys and girls in most food groups (Table 2). Nevertheless, significant differences between the sexes were observed in three food groups. The correlation coefficients for vegetables and sport/energy drinks in the female study group were significantly lower and the correlation coefficient for poultry significant higher than in the male group. Compared to younger participants, adolescents aged 16 to 17 showed a tendency to higher correlation coefficients. Significantly higher correlation coefficients were observed for 16- to 17-year-olds than for younger adolescents for fish and white bread. In addition, 16- to 17-year-old adolescents had significantly higher coefficients for coffee and breakfast cereals than 12- to 13-year-olds, and higher coefficients for fast food than 14- to 15-year-olds. Table 3 shows the correlation coefficients between the mean daily food intake assessed with the FFQ and the DISHES interview stratified for relative bodyweight (normal weight, overweight) and socio-economic status (low, medium, high). Coefficients for overweight adolescents were lower than those for adolescents with normal body weight in most cases. Significant differences were observed in the case of fruit/herbal tea, butter, cream cheese, meat products, cakes/pastries, sweets, and hazelnut spread; correlation coefficients were higher among normal-weight respondents. After additionally stratifying for sex, a tendency towards lower correlation coefficients was observed among overweight girls compared to normal-weight girls, while overweight boys often showed higher coefficients than normal-weight boys (see Additional file 2, Table S1). A comparison between adolescents with low and high socio-economic status showed a tendency towards higher coefficients for adolescents with higher status (Table 4). Significant differences between these groups were found in the case of milk, fruit/herbal tea, breakfast cereals, meat products, potatoes, fast food, ketchup/mayonnaise, and cakes/pastries; correlation was higher for high socio-economic status. A further stratification for sex showed similar results for boys and girls (see Additional file 2, Table S2).

**Table 3:** Correlation coefficients (95% CI) of food group intake between both methods in subgroups*

Food group	Body weight^a^		Socio-economic status^b^		
	
	normal weight n = 1005	overweight n = 201	low n = 249	medium n = 617	high n = 331
Milk	0.67 (.63-.70)	0.65 (.56-.72)	**0.58 (.49-.66)**	0.66 (.61-.70)	**0.74 (.69-.79)**
Soda	0.67 (.63-.70)	0.54 (.43-.63)	0.62 (.54-.69)	0.63 (.58-.68)	0.67 (.61-.73)
Sport/energy drinks	0.33 (.27-.38)	0.17 (.03-.30)	0.19 (.07-.31)	0.32 (.25-.39)	0.38 (.28-.47)
Juice	0.56 (.52-.60)	0.46 (.34-.56)	0.44 (.33-.54)	0.56 (.50-.61)	0.58 (.50-.65)
Tap water	0.44 (.39-.49)	0.35 (.22-.47)	0.34 (.23-.45)	0.41 (.34-.47)	0.49 (.40-.57)
Mineral water	0.67 (.63-.70)	0.56 (.46-.65)	0.67 (.60-.73)	0.67 (.62-.71)	0.62 (.55-.68)
Fruit/herbal tea	**0.64 (.60-.68)**	**0.47 (.35-.57)**	**0.47 (.37-.56)**	**0.62 (.57-.67)**	**0.71 (.65-.76)**
Green/black tea	0.61 (.57-.65)	0.64 (.55-.72)	0.69 (.62-.75)	0.57 (.51-.62)	0.61 (.54-.67)
Coffee	0.68 (.65-.71)	0.67 (.59-.74)	0.64 (.56-.71)	0.65 (.60-.69)	0.75 (.70-.79)
Breakfast cereals	0.66 (.62-.69)	0.66 (.57-.73)	**0.50 (.40-.59)**	**0.69 (.65-.73)**	**0.73 (.68-.78)**
Brown bread^1^	0.36 (.30-.41)	0.30 (.17-.42)	0.29 (.17-.40)	0.35 (.28-.42)	0.39 (.29-.48)
White bread^2^	0.31 (.25-.36)	0.33 (.20-.45)	0.33 (.21-.44)	0.28 (.21-.35)	0.35 (.25-.44)
Butter	**0.64 (.60-.68)**	**0.49 (.38-.59)**	0.42 (.31-.52)	0.65 (.60-.69)	0.70 (.64-.75)
Margarine	0.69 (.66-.72)	0.67 (.59-.74)	0.65 (.57-.72)	0.68 (.64-.72)	0.73 (.68-.78)
Cheese	0.63 (.59-.67)	0.51 (.40-.61)	0.49 (.39-.58)	0.64 (.59-.68)	0.63 (.56-.69)
Curd^3^	0.56 (.52-.60)	0.53 (.42-.62)	0.46 (.36-.55)	0.58 (.53-.63)	0.59 (.51-.66)
Cream cheese	**0.61 (.57-.65)**	**0.43 (.31-.54)**	0.59 (.50-.67)	0.55 (.49-.60)	0.65 (.58-.71)
Eggs	0.59 (.55-.63)	0.57 (.47-.66)	0.58 (.49-.66)	0.58 (.53-.63)	0.59 (.51-.66)
Soup	0.50 (.45-.54)	0.61 (.52-.69)	0.48 (.38-.57)	0.49 (.43-.55)	0.60 (.53-.66)
Meat	0.46 (.41-.51)	0.50 (.39-.60)	0.41 (.30-.51)	0.45 (.38-.51)	0.55 (.47-.62)
Poultry	0.41 (.36-.46)	0.33 (.20-.45)	0.36 (.25-.46)	0.40 (.33-.46)	0.43 (.34-.51)
Meat products	**0.58 (.54-.62)**	**0.42 (.30-.53)**	**0.46 (.36-.55)**	0.55 (.49-.60)	**0.63 (.56-.69)**
Fish	0.60 (.56-64)	0.62 (.53-.70)	0.56 (.47-.64)	0.57 (.51-.62)	0.67 (.61-.73)
Fruits	0.64 (.60-.68)	0.58 (.48-.67)	0.55 (.46-.63)	0.67 (.62-.71)	0.60 (.53-.66)
Vegetables	0.45 (.40-.50)	0.40 (.28-.51)	0.40 (.29-.50)	0.45 (.38-.51)	0.51 (.43-.59)
Pasta/rice	0.21 (.15-.27)	0.29 (.16-.41)	0.14 (.02-.26)	0.21 (.13-.28)	0.29 (.19-.39)
Potatoes	0.56 (.52-.60)	0.43 (.31-.54)	**0.42 (.31-.52)**	0.54 (.48-.59)	**0.60 (.53-.66)**
Potato products	0.48 (.43-.53)	0.50 (.39-.60)	0.42 (.31-.52)	0.49 (.43-.55)	0.52 (.44-.59)
Fast food^4^	0.53 (.48-.57)	0.51 (.40-.61)	**0.42 (.31-.52)**	0.51 (.45-.57)	**0.60 (.53-.66)**
Ketchup/mayonnaise	0.55 (.51-.59)	0.62 (.53-.70)	**0.39 (.28-.49)**	0.58 (.53-.63)	**0.63 (.56-.69)**
Cakes/pastries	**0.47 (.42-.52)**	**0.28 (.15-.40)**	**0.36 (.25-.46)**	0.43 (.36-.49)	**0.56 (.48-.63)**
Cookies	0.41 (.36-.46)	0.43 (.31-.54)	0.39 (.28-.49)	0.43 (.36-.49)	0.39 (.29-.48)
Sweets^5^	**0.54 (.49-.58)**	**0.36 (.23-.47)**	0.48 (.38-.57)	0.54 (.48-.59)	0.49 (.40-.57)
Ice cream	0.61 (.57-.65)	0.50 (.39-.60)	0.53 (.43-.61)	0.61 (.56-.66)	0.59 (.51-.66)
Pudding/rice pudding	0.50 (.45-.54)	0.47 (.35-.57)	0.38 (.27-.48)	0.53 (.47-.58)	0.55 (.47-.62)
Pancakes	0.50 (.45-.54)	0.43 (.31-.54)	0.42 (.31-.52)	0.48 (.42-.54)	0.58 (.50-.65)
Sweet spreads	0.65 (.61-.68)	0.66 (.57-.73)	0.63 (.55-.70)	0.63 (.58-.68)	0.71 (.65-.76)
Hazelnut spread	**0.68 (.65-.71)**	**0.50 (.39-.60)**	0.58 (.49-.66)	0.69 (.65-.73)	0.71 (.65-.76)
Salty snacks	0.57 (.53-.61)	0.56 (.46-.65)	0.50 (.40-.59)	0.56 (.50-.61)	0.63 (.56-.69)
Nuts	0.36 (.30-.41)	0.46 (.34-.56)	0.31 (.19-.42)	0.37 (.30-.44)	0.45 (.36-.53)

Mean	0.54	0.49	0.46	0.53	0.58

Abbreviation: CI (confidence interval)

^a ^According to Kromeyer-Hauschild et al. [24]

^b ^According to Winkler [22]

*Non-overlapping 95% confidence intervals (bold) were considered statistically significant

^1^Brown bread, brown bun

^2^White bread, white bun

^3^Curd, yoghurt, soured milk

^4^Burger, doner kebab, fried/grilled sausage, curried sausage

^5^Sweets, fruit chews, chocolate

**Table 4:** Mean of food intake assessed by the FFQ and mean of difference between both methods

Food group	n	FFQ		Mean difference*	Mean difference%**	P2.5-P97.5 of mean difference
		Mean	SD			
Milk	1205	230.2	355.8	3.1	1.4	-406.1-738.5
Soda	1205	383.9	698.6	-44.8	-11.0	-1518.8-1608.4
Sport/energy drinks	1201	26.0	110.7	11.5	56.8	-85.5-200
Juice	1200	306.3	526.9	-172.6	-44.0	-1456.8-964.1
Tap water	1204	246.3	622.4	142.3	81.2	-599.1-2090
Mineral water	1206	636.2	922.0	-60.3	-9.0	-1845-2248.8
Fruit/herbal tea	1203	75.1	206.1	-23.7	-27.2	-522.9-277.9
Green/black tea	1207	33.4	182.2	8.1	27.6	-116.8-150
Coffee	1208	24.3	87.7	-6.4	-23.2	-159.5-113
Breakfast cereals	1207	21.5	33.8	-3.7	-15.8	-89.8-66.6
Brown bread^1^	1206	64.0	95.1	9.5	16.0	-190-250
White bread^2^	1210	66.6	86.8	-30.1	-36.9	-197.3-187.2
Butter	1193	5.2	9.4	-4.7	-62.3	-43.1-18.4
Margarine	1206	4.0	8.3	-5.6	-82.4	-45.3-10
Cheese	1203	24.9	40.2	5.9	26.9	-49.2-111
Curd^3^	1206	73.5	103.9	14.2	21.4	-137.2-200
Cream cheese	1200	6.5	19.2	1.4	24.3	-18.6-30
Eggs	1200	13.2	22.5	0.3	2.3	-32.8-40
Soup	1209	75.4	118.6	8.0	11.2	-181.4-185.7
Meat	1202	43.2	60.6	3.3	8.0	-86.4-113.6
Poultry	1199	24.1	33.1	8.9	45.3	-38-81.3
Meat products	1200	20.4	24.5	-26.3	-78.3	-119.3-30.6
Fish	1210	10.8	17.9	-0.3	-2.7	-34.4-30.9
Fruits	1207	214.7	304.1	12.9	6.2	-392.9-650.5
Vegetables	1192	137.3	158.2	-36.8	-23.6	-370.2-263.6
Pasta/rice	1208	52.4	61.2	35.0	100.3	-36.8-183.3
Potatoes	1207	79.6	79.0	18.0	25.5	-102.7-196.9
Potato products	1209	21.4	45.5	0.4	1.9	-62.1-64.3
Fast food^4^	1209	22.7	72.2	-11.3	-39.8	-110.4-41.8
Ketchup/mayonnaise	1205	4.2	9.9	-1.5	-30.0	-25.5-14.2
Cakes/pastries	1208	25.1	36.2	6.1	27.7	-52.9-97.5
Cookies	1203	5.8	10.5	-1.1	-17.5	-36.7-23.5
Sweets^5^	1201	28.8	53.5	-10.0	-29.6	-97.8-70.3
Ice cream	1205	34.2	100.2	22.0	95.0	-20.5-150
Pudding/rice pudding	1212	17.1	37.1	-6.6	-32.4	-94.9-52.4
Pancakes	1211	12.7	55.0	3.7	34.1	-30.1-35.7
Sweet spreads	1208	5.1	8.9	-3.0	-45.8	-33.1-14.1
Hazelnut spread	1205	6.0	12.4	-3.3	-43.1	-40-13.7
Salty snacks	1211	10.0	30.8	2.4	27.3	-24.4-36.8
Nuts	1210	1.3	5.3	-1.2	-64.9	-18.9-5.4

Abbreviation: FFQ (food frequency questionnaire), P (percentile), SD (standard deviation)

*Calculated according to formula: mean of difference = Mean (FFQ - DISHES);

**Calculated using the formula: $\text{Difference}\;\left(\%\right)=\frac{\text{Mean}\;\left(\text{FFQ}-\text{DISHES}\right)}{\left(\text{Mea}{\text{n}}_{\text{FFQ}}+\text{Mea}{\text{n}}_{\text{DISHES}}\right)∕2}*100$

^1^Brown bread, brown bun

^2^White bread, white bun

^3^Curd, yoghurt, soured milk

^4^Burger, doner kebab, fried/grilled sausage, curried sausage

^5^Sweets, fruit chews, chocolate

### Ranking classification

Table 5 presents the agreement between quartiles of food consumption from the FFQ and quartiles from the DISHES interview. The proportion of participants classified in the same and adjacent quartile varied between 70.1% for pasta/rice and 90.8% for coffee. Classification in opposite quartiles varied between 1.9% for soda/mineral water and 9.7% for tap water. For most food groups Cohen's weighted kappa showed values between 0.21 and 0.60. Only the food groups white bread and pasta/rice showed values below 0.20.

**Table 5:** Agreement of quartiles for food group intake assessed by both methods

Food group	Same (%)	Adjacent (%)	Opposite (%)	Weighted kappa
Quartile				

Mineral water	51,1	37,5	1,9	0,494
Milk	49,5	38,6	2,0	0,472
Soda	48,1	40,1	1,9	0,471
Fruits	47,2	40,4	2,5	0,455
Cheese	46,8	37,4	2,7	0,445
Salty snacks	44,3	38,0	3,5	0,396
Curd^1^	42,9	42,3	2,1	0,393
Ketchup/mayonnaise	43,4	41,5	2,7	0,391
Potatoes	45,3	37,4	3,4	0,389
Meat products	42,4	40,2	2,8	0,385
Fast food^2^	43,8	41,9	2,4	0,374
Soup	42,0	38,1	3,4	0,367
Potato products	40,9	39,1	3,5	0,351
Sweets^3^	41,2	39,1	2,2	0,350
Cakes/pastries	41,1	38,2	4,0	0,320
Cookies	40,1	37,7	6,1	0,301
Vegetables	38,9	39,3	4,5	0,300
Meat	39,5	36,5	5,9	0,299
Poultry	37,9	36,8	7,3	0,273
Juice	44,7	38,3	4,3	0,202
White bread^4^	33,1	40,2	6,4	0,192

Adapted groups*				

Coffee	75,2	15,6	3,1	0,589
Margarine	55,1	35,0	2,3	0,543
Black/green tea	77,4	13,2	2,7	0,499
Hazelnut spread	50,8	35,6	2,6	0,497
Sweet spreads	49,0	38,4	3,1	0,482
Breakfast cereals	47,9	40,7	2,0	0,479
Butter	49,7	37,0	4,2	0,477
Fruit/herbal tea	51,0	32,6	4,9	0,454
Fish	48,3	36,2	3,4	0,451
Ice cream	44,1	40,0	3,5	0,419
Eggs	44,8	41,5	2,3	0,410
Cream cheese	50,4	29,2	5,8	0,399
Pudding/rice pudding	45,8	33,9	5,4	0,379
Pancakes	46,7	31,4	8,2	0,371
Nuts	54,2	21,8	9,3	0,295
Tap water	53,6	22,7	9,7	0,293
Brown bread^5^	37,6	37,1	8,4	0,250
Sport/energy drinks	66,1	13,5	8,8	0,202
Pasta/rice	31,1	39,0	9,6	0,151

*Since more than 25% of participants reported no consumption, these were defined as one group and the remaining participants were grouped into tertiles.

^1^Curd, yoghurt, soured milk

^2^Burger, doner kebab, fried/grilled sausage, curried sausage

^3^Sweets, fruit chews, chocolate

^4^White bread, white bun

^5^Brown bread, brown bun

### Mean differences

Table 4 shows the mean food-group intakes per day estimated by the two methods and the 2.5 and 97.5 percentiles of the differences. The mean difference ranged from 1.4% for milk to 100.3% for pasta/rice. Milk, mineral water, eggs, meat, fish, fruits and potato products showed differences of less than 10%. Food consumption as assessed by the FFQ was not generally higher or lower than the consumption estimated by the DISHES interview. The intake of soda, juice, mineral water, fruit/herbal tea, coffee, breakfast cereals, white bread, butter, margarine, meat products, fish, vegetables, fast food, ketchup/mayonnaise, cookies, sweets, pudding/rice pudding, sweet spreads, hazelnut spread, and nuts assessed by the FFQ was lower than the estimates by the DISHES interview. The intake of milk, sport/energy drinks, tap water, black/green tea, brown bread, cheese, curd, cream cheese, eggs, soup, meat, poultry, fruits, pasta/rice, potatoes, potato products, cakes/pastries, ice cream, pancakes, and salty snacks was higher. The 2.5 percentiles and 97.5 percentile of differences covered a wide range.

## Discussion

In the present study, the validity of the KiGGS FFQ was evaluated in comparison to a diet history method instrument. Due to measurement errors and limitations within every dietary assessment method, only relative validity can be determined. The FFQ showed a fair to moderate agreement in ranking participants towards their intake for most food groups compared to the DISHES interview [27]. Only white bread and pasta/rice showed slight agreement. The correlation coefficients varied between 0.22 for pasta/rice and 0.69 for margarine. A reasonable to good correlation between the two instruments was found for 67% of the food groups [28]. The average of the observed correlation coefficients was higher or equal to other FFQ validation studies for adolescents [6-9,29-32]. The observed correlation coefficients are also similar to results from FFQ validation studies for adults [33-36]. Individual, higher coefficients for adults may be caused by an established meal structure and therefore a better memory on portion size and frequency. By contrast, the food frequency and portion sizes of adolescents are not constant [37]. Agreement of mean intake is rather low in most food groups. Some food groups - like milk, mineral water, eggs, meat fish, fruits and potato products - show small average differences. However, on the individual level there is a wide range of differences for every food group. The FFQ should therefore perhaps not be used to estimate absolute intakes. Other youth validation studies on food group level came to similar results [9,29].

The validation was performed using food consumption data from the EsKiMo module. This offered the advantage of a large validation sample that is representative of German adolescents, which also made it possible to evaluate the validity in subgroups. However, there may have been a tendency to select participants who were especially interested in their health and nutrition, since the EsKiMo participants agreed to participate for a second time. Calculation of correlation coefficients is a common method in validation studies [38]. One main reason may be that it facilitates comparisons with other study results [39]. However, correlation coefficients only measure the strength of the association between two methods, not the agreement, and can be a misleading indicator of validity [40,41]. Nevertheless, calculating correlation coefficients was included in this study since small correlation coefficients can be indicators of potential error sources [42]. Additional analyses, like Bland Altman analysis or ranking classification, can avoid misleading conclusions. For the Bland Altman analysis it is assumed that the differences between two measurements are normally distributed [43]. Since in our study the differences were not normally distributed, and this could not be improved by log-transformation, the differences between the two instruments were calculated on the basis of untransformed data. Furthermore, we included an adapted analysis, which approximates the analysis of limits of agreement. Percentiles (2.5/97.5) of differences between the methods were calculated, which also represents 95% of differences. There are some limitations to be considered in relation to this validation study. For the assessment of validity, the reference method should have independent error sources [44]. Contrary to this, the reference instrument DISHES also relies on the memories of the participants and their perceptions of portion sizes, like the FFQ. This may result in unrealistically higher estimates of validity. Since the EsKiMo study was not primarily designed as a validation study, the choice of another reference method was not applicable. However, the DISHES interviews were conducted by trained nutritionists and supported by standardized software, while the FFQ was self-administered. Dietary intake information was more detailed and assessed in a meal-based structure. The DISHES interview used a variety of tableware models, standard portions and a picture book for estimating portion sizes. Furthermore, the list of food items assessed by the FFQ was fixed, while the DISHES interview was open-ended. The DISHES interview therefore seems an acceptable method of comparison. The DISHES method was previously validated for adults, but not for adolescents. It has also been used in some large nutrition surveys. Nevertheless, a pre-test was conducted to test feasibility among adolescents and the food-code database was adopted for younger persons. The FFQ was filled in by respondents several days before the DISHES interview was conducted. The sequence of instruments is relevant, since one measurement may affect a later response [44]. However, the reverse sequence would probably have a larger effect, since the diet history is a more comprehensive instrument which may have a larger impact on a person's memory and awareness of the actual diet. In addition, the items in DISHES are more detailed and asked in a face-to-face setting. We therefore think the influence on recall of the applied sequence is minor.

The FFQ seems to be suitable for all considered subpopulations, since most food groups showed reasonable to acceptable correlation coefficients. Only the groups pasta/rice, white/brown bread, and cakes/pastries showed correlation coefficients of below 0.3; these should be interpreted with caution. Despite these results, certain differences between the BMI and socio-economic groups were found. As expected, older participants (aged 16 to 17 years) showed a tendency towards higher correlation coefficients than younger ones (12 to 13 years), because their cognitive abilities were better developed [37]. Furthermore, older adolescents choose their food themselves more often; they are also more conscious of what they eat. Correlation coefficients were lower for overweight adolescent girls compared to normal-weight girls. This finding might be expected, since thinness and body image have an important influence on female adolescents' dietary reporting [45]. Boys are less likely to be concerned about their body image. This relationship is in line with results from other studies [46,47]. In addition, participants who live in families with a low socio-economic status showed lower correlations more often than participants in families with high socio-economic status. To our knowledge, similar studies in such subgroups have not been performed among adolescents. However, some studies among adults found an inverse association between socio-economic status and underreporting [48,49], which is a potential source of bias in nutritional epidemiology and could be one reason for lower reporting validity. Nevertheless, the difference between subgroups is marginal and most food groups showed acceptable to good correlations. The KiGGS-FFQ is thus also suitable for groups with lower socio-economic status and higher body mass index.

Some differences in ranking and mean estimates between the instruments may be caused by differences in the measurement of portion sizes. While the DISHES interview assesses food intake with a variety of standard portions, tableware models and a picture book, the FFQ uses predefined, simple categories. The variability of values measured by the FFQ is therefore rather low. The relatively weak ranking agreement in the case of vegetables is discussed in other studies among adolescents [9,29], and also in adult populations [33,35,50]. One possible explanation is again related to difficulties in estimating portion size in some food groups. Some broad food items like "cooked", "frozen", "tinned", and "raw vegetables" may complicate the estimation of these predefined portion sizes. For instance, lettuce and tomatoes both belong to the raw vegetables group, even though one portion of each may have very different weights. In addition, adolescents in particular may have problems defining the origin of their foods, because they normally do not prepare meals themselves. Accordingly, dividing vegetables into the groups frozen and tinned seems difficult for this age group. This difference was not assessed in the DISHES interview. These items were therefore grouped. The food group pasta/rice showed slight agreement among ranking participants in terms of food intake. One possible explanation is the different use of the two products. Pasta is often the main component of a meal like spaghetti bolognaise, while rice is eaten as a side dish. It is therefore difficult to predefine a portion size for both products together. The DISHES interview assesses the amounts separately for every food and in as much detail as possible. The group white bread also showed only slight agreement in ranking. This may be due to a lack of experience among adolescents regarding the classification of bread.

## Conclusions

The FFQ shows fair to moderate ranking validity for most food groups except pasta/rice and white bread. Estimates for these two food groups should be interpreted with caution. As for the complete diet, the ability to assess absolute intakes using the FFQ is limited; but also for single foods there is no evidence whether the data of the DISHES interview or the FFQ are closer to the truth. Overall, the relative validity of the KiGGS FFQ is comparable to FFQs from the current literature [9,29,33,35,36]. The FFQ seems suitable for collecting representative dietary data at the population level, which allows exposure comparison and confounder adjustments. Based on correlation coefficients, the validity is similar for age, sex, body weight, and socio-economic status subgroups.

## List of abbreviations used

BMI: Body mass index; CI: Confidence interval; DISHES: Dietary Interview Software for Health Examination Studies; EsKiMo: Eating Study as a KiGGS Module; FFQ: Food Frequency Questionnaire; KiGGS: German Health Interview and Examination Survey for Children and Adolescents; P: Percentile; SD: Standard deviation.

## Competing interests

The authors declare that they have no competing interests.

## Authors' contributions

GBMM was one of the designers and the project leader of EsKiMo; AR organized the field work and data handling of EsKiMo; JT conducted the presented analysis and drafted the manuscript; GBMM and AR assisted with statistical analysis, interpreting the results and writing the manuscript. GM is the main responsible developer of DISHES and of the FFQ. AR was involved in the development of DISHES. All the authors were involved in the critical revision of the manuscript for important intellectual content. All authors read and approved the final manuscript.

## Supplementary Material

Additional file 1 **Recoding of frequency and portion size data**. The file contains information about the recoding of the frequencies and the portion sizes of each food item to calculate the average food-group intake per day. Click here for file

Additional file 2 **Correlation coefficients between both methods by body weight, socio-economic status and sex**. The file contains the results of correlation analyses of food group intake between both instruments for subgroups according to body weight, socio-economic status and sex. Click here for file
