# Supplementary material for: Relative validation of the KiGGS Food Frequency Questionnaire among adolescents in Germany
Source: Nutr J. 2011 Dec 7;10:133. doi: 10.1186/1475-2891-10-133 (PMC3261099; doi:10.1186/1475-2891-10-133)
Supplement: Additional file 1 — Recoding of frequency and portion size data. The file contains information about the recoding of the frequencies and the portion sizes of each food item to calculate the average food-group intake per day. [file 1475-2891-10-133-S1.PDF]

Additional file 1: Recoding of frequency and portion size data

Table 1: Weighting values used for recoding of frequencies

Category		Value
1	Never	0
2	once a month	1
3	two to three times a month	2.5
4	once or twice a week	6
5	three to four times a week	14
6	five to six times a week	22
7	once a day	28
8	two to three times a day	70
9	four to five times a day	126
10	more than five times a day	168

Table 2: Portion sizes used for calculation of average food intake

FFQ question	Food item	Average portion size (category 3)	Amount in gram
1a	Milk	1 glass (200 ml)	200
2a	Soda	1 glass (200 ml)	200
3a	Sport/energy drinks	1 glass (200 ml)	200
4a	Juice	1 glass (200 ml)	200
5a	Tap water	1 glass (200 ml)	200
6a	Mineral water	1 glass (200 ml)	200
7a	Fruit/herbal tea	1 cup (150 ml)	150
8a	Green/black tea	1 cup (150 ml)	150
9a	Coffee	1 cup (150 ml)	150
10a	Breakfast cereals	1 bowl	50
11a	Brown bread	1 slice/bun	50
12a	White bread	1 slice/bun	50
13a	Butter	2 teaspoon	10
14a	Margarine	2 teaspoon	10
15a	Cheese	1 slice/portion	30
16a	Curd, yoghurt, soured milk	1 cup	200
17a	Cream cheese	2 spoon	30
18a	Eggs	1 egg	60
19a	Soup	1 dish	400
20a	Meat	0.5 portion	60
21a	Poultry	0.5 portion	75
22a	Meat products	1 slice	20
23a	Fish	1 portion	90
24a	Raw fruits	1 piece/bowl	150
25a	Tinned fruits	1 bowl	150
26a	Cooked vegetables	1 portion	150
27a	Frozen vegetables	1 portion	150
28a	Tinned vegetables	1 portion	150
29a	Raw vegetables	1 portion	100
29b	Salad dressing		1 spoon = 12 g
30a	Pasta/rice	1 dish	125
31a	Potatoes	1 portion/2 potatoes	175
32a	Potato products	1 portion	150
33a	Fast food	1 portion<br>(meat within 1 portion)	195<br>(85)
34a	Ketchup/mayonnaise	2 spoon	20
35a	Cakes/pastries	1 piece	100
36a	Cookies	3 cookies	15
37a	Chocolate	0.5 chocolate bar	50
38a	Sweets	6-10 piece	16
39a	Ice cream	2 scoops	150
40a	Pudding/rice pudding	1 bowl	150
41a	Pancakes	1 piece	100
42a	Sweet spreads	2 teaspoon	20
43a	Hazelnut spread	2 teaspoon	20
44a	Salty snacks	1 bowl	50
45a	Nuts	1 portion	25

Average food consumption per day = Amount * Frequency value / 28
