# Supplementary material for: Relative validation of the KiGGS Food Frequency Questionnaire among adolescents in Germany
Source: Nutr J. 2011 Dec 7;10:133. doi: 10.1186/1475-2891-10-133 (PMC3261099; doi:10.1186/1475-2891-10-133)
Supplement: Additional file 2 — Correlation coefficients between both methods by body weight, socio-economic status and sex. The file contains the results of correlation analyses of food group intake between both instruments for subgroups according to body weight, socio-economic status and sex. [file 1475-2891-10-133-S2.PDF]

Additional file 2: Correlation coefficients between both methods by body weight, socio-economic status and sex

Table 1: Correlation coefficients (95% CI) of food group intake between both methods by body weight[a] and sex*

Food group	Male		Female	
	normal weight n=477	overweight n=101	normal weight n=528	overweight n=100
Milk	0.73 (.69-.77)	0.65 (.52-.75)	0.67 (.62-.71)	0.55 (.40-.67)
Soda	0.54 (.47-.60)	0.70 (.58-.79)	0.62 (.56-.67)	0.51 (.35-.64)
Sport/energy drinks	0.25 (.16-.33)	0.40 (.22-.55)	0.20 (.12-.28)	0.05 (-.15-.24)
Juice	0.39 (.31-.46)	0.56 (.41-.68)	0.56 (.50-.62)	0.52 (.36-.65)
Tap water	0.37 (.29-.45)	0.45 (.28-.59)	0.43 (.36-.50)	0.32 (.13-.49)
Mineral water	0.51 (.44-.57)	0.69 (.57-.78)	0.63 (.58-.68)	0.62 (.48-.73)
Fruit/herbal tea	0.46 (.39-.53)	0.63 (.50-.73)	0.64 (.59-.69)	0.47 (.30-.61)
Green/black tea	0.58 (.52-.64)	0.58 (.43-.70)	0.64 (.59-.69)	0.72 (.61-.80)
Coffee	0.58 (.52-.64)	0.69 (.57-.78)	0.68 (.63-.72)	0.75 (.65-.82)
Breakfast cereals	0.65 (.59-.70)	0.67 (.55-.77)	0.64 (.59-.69)	0.66 (.53-.76)
Brown bread[1]	0.34 (.26-.42)	0.36 (.18-.55)	0.35 (.27-.42)	0.26 (.07-.43)
White bread[2]	0.24 (.15-.32)	0.23 (.04-.41)	0.32 (.24-.39)	0.30 (.11-.47)
Butter	**0.42 (.34-.49)**	**0.65 (.52-.75)**	0.62 (.56-.67)	0.55 (.40-.67)
Margarine	0.64 (.58-.69)	0.64 (.51-.74)	0.73 (.69-.77)	0.72 (.61-.80)
Cheese	0.52 (.45-.58)	0.61 (.47-.72)	0.64 (.59-.69)	0.52 (.36-.65)
Curd[3]	0.51 (.44-.57)	0.55 (.40-.67)	0.58 (.52-.63)	0.55 (.40-.67)
Cream cheese	0.34 (.26-.42)	0.55 (.40-.67)	0.64 (.59-.69)	0.51 (.35-.64)
Eggs	0.47 (.40-.54)	0.55 (.40-.67)	0.59 (.53-.64)	0.63 (.49-.74)
Soup	0.51 (.44-.57)	0.51 (.35-.64)	0.49 (.42-.55)	0.70 (.58-.79)
Meat	0.46 (.39-.53)	0.39 (.21-.54)	0.47 (.40-.53)	0.49 (.32-.63)
Poultry	0.28 (.20-.36)	0.31 (.12-.48)	0.48 (.41-.54)	0.39 (.21-.54)
Meat products	0.34 (.26-.42)	0.51 (.35-.64)	0.60 (.54-.65)	0.44 (.27-.59)
Fish	0.50 (.43-.56)	0.58 (.43-.70)	0.61 (.55-.66)	0.73 (.62-.81)
Fruits	0.58 (.52-.64)	0.63 (.50-.73)	0.63 (.58-.68)	0.57 (.42-.69)
Vegetables	0.53 (.46-.59)	0.46 (.29-.60)	0.42 (.35-.49)	0.24 (.05-.42)
Pasta/rice	0.34 (.26-.42)	0.15 (-.05-.34)	0.26 (.18-.34)	0.25 (.06-.43)
Potatoes	0.36 (.28-.44)	0.57 (.42-.69)	0.55 (.49-.61)	0.51 (.35-.64)
Potato products	0.45 (.38-.52)	0.46 (.29-.60)	0.50 (.43-.56)	0.52 (.36-.65)
Fast food[4]	0.38 (.30-.45)	0.52 (.36-.65)	0.46 (.39-.52)	0.53 (.37-.66)
Ketchup/mayonnaise	0.62 (.56-.67)	0.56 (.41-.68)	0.53 (.47-.59)	0.59 (.45-.70)
Cakes/pastries	0.37 (.29-.45)	0.45 (.28-.59)	**0.48 (.41-.54)**	**0.16 (-.04-.35)**
Cookies	0.45 (.38-.52)	0.43 (.26-.58)	0.39 (.32-.46)	0.42 (.24-.57)
Sweets[5]	0.35 (.27-.43)	0.53 (.37-.66)	0.55 (.49-.61)	0.36 (.18-.52)
Ice cream	0.56 (.50-.62)	0.57 (.42-.69)	**0.66 (.61-.71)**	**0.45 (.28-.59)**
Pudding/rice pudding	0.48 (.41-.55)	0.50 (.34-.63)	0.50 (.43-.56)	0.46 (.29-.60)
Pancakes	0.38 (.30-.45)	0.49 (.33-.63)	0.51 (.44-.57)	0.46 (.29-.60)
Sweet spreads	0.63 (.57-.68)	0.65 (.52-.75)	0.66 (.61-.71)	0.70 (.58-.79)
Hazelnut spread	0.44 (.36-.51)	0.63 (.50-.73)	**0.73 (.69-.77)**	**0.53 (.37-.66)**
Salty snacks	0.65 (.59-.70)	0.57 (.42-.69)	0.57 (.51-.62)	0.43 (.26-.58)
Nuts	0.54 (.47-.60)	0.34 (.15-.50)	0.39 (.32-.46)	0.36 (.18-.52)
Mean	0.47	0.52	0.54	0.49

Abbreviation: CI (confidence interval)
[a]According to Kromeyer-Hauschild et al. [23]
*Non-overlapping 95% confidence intervals (bold) were considered statistical significant
[1]Brown bread, brown bun
[2]White bread, white bun
[3]Curd, yoghurt, soured milk
[4]Burger, doner kebab, fried/grilled sausage, curried sausage
[5]Sweets, fruit chews, chocolate

Table 2: Correlation coefficients (95% CI) of food group intake between both methods by socio-economic status[a] and sex*

Food group	Male		Female	
	low SES n=115	high SES n=166	low SES n=134	high SES n=165
Milk	0.59 (.46-.70)	0.74 (.66-.80)	**0.51 (.37-.63)**	**0.75 (.67-.81)**
Soda	0.63 (.51-.73)	0.66 (.56-.74)	0.58 (.46-.68)	0.68 (.59-.75)
Sport/energy drinks	0.31 (.13-.47)	0.45 (.32-.56)	0.03 (-.14-.20)	0.27 (.12-.41)
Juice	0.42 (.26-.56)	0.53 (.41-.63)	0.45 (.30-.58)	0.63 (.53-.71)
Tap water	0.40 (.23-.54)	0.49 (.36-.60)	0.29 (.13-.44)	0.49 (.36-.60)
Mineral water	0.62 (.49-.72)	0.65 (.55-.73)	0.70 (.60-.78)	0.59 (.48-.68)
Fruit/herbal tea	0.43 (.27-.57)	0.66 (.56-.74)	**0.50 (.36-.62)**	**0.75 (.67-.81)**
Green/black tea	0.65 (.53-.74)	0.52 (.40-.62)	0.72 (.63-.79)	0.69 (.60-.76)
Coffee	0.60 (.47-.71)	0.76 (.69-.82)	0.68 (.58-.76)	0.75 (.67-.81)
Breakfast cereals	**0.47 (.31-.60)**	**0.70 (.61-.77)**	0.52 (.38-.63)	0.71 (.63-.78)
Brown bread[1]	0.35 (.18-.50)	0.38 (.24-.50)	0.23 (.06-.38)	0.41 (.27-.53)
White bread[2]	0.27 (.09-.43)	0.29 (.14-.42)	0.25 (.08-.40)	0.34 (.20-.47)
Butter	**0.35 (.18-.50)**	**0.73 (.65-.79)**	0.49 (.35-.61)	0.67 (.58-.75)
Margarine	0.65 (.53-.74)	0.66 (.56-.74)	**0.62 (.50-.71)**	**0.79 (.72-.84)**
Cheese	0.56 (.42-.67)	0.59 (.48-.68)	**0.43 (.28-.56)**	**0.67 (.58-.75)**
Curd[3]	0.42 (.26-.56)	0.58 (.47-.67)	0.50 (.36-.62)	0.59 (.48-.68)
Cream cheese	0.46 (.30-.59)	0.62 (.52-.71)	0.69 (.59-.77)	0.64 (.54-.72)
Eggs	0.52 (.37-.64)	0.54 (.42-.64)	0.60 (.48-.70)	0.61 (.50-.70)
Soup	0.47 (.31-.60)	0.61 (.50-.70)	0.50 (.36-.62)	0.60 (.49-.69)
Meat	0.31 (.13-.47)	0.48 (.35-.59)	0.42 (.27-.55)	0.53 (.41-.63)
Poultry	0.21 (.03-.38)	0.35 (.21-.48)	0.45 (.30-.58)	0.51 (.39-.61)
Meat products	0.51 (.36-.63)	0.54 (.42-.64)	**0.42 (.27-.55)**	**0.67 (.58-.75)**
Fish	0.51 (.36-.63)	0.62 (.52-.71)	0.61 (.49-.71)	0.66 (.56-.74)
Fruits	0.54 (.40-.66)	0.63 (.53-.71)	0.57 (.44-.67)	0.59 (.48-.68)
Vegetables	0.48 (.33-.61)	0.54 (.42-.64)	0.31 (.15-.46)	0.48 (.35-.59)
Pasta/rice	0.19 (.01-.36)	0.30 (.15-.43)	0.09 (-.08-.26)	0.29 (.14-.42)
Potatoes	0.44 (.28-.58)	0.62 (.52-.71)	0.40 (.25-.53)	0.59 (.48-.68)
Potato products	0.45 (.29-.58)	0.51 (.39-.61)	0.39 (.24-.52)	0.51 (.39-.61)
Fast food[4]	0.51 (.36-.63)	0.57 (.46-.66)	0.29 (.13-.44)	0.52 (.40-.62)
Ketchup/mayonnaise	**0.31 (.13-.47)**	**0.68 (.59-.75)**	0.44 (.29-.57)	0.54 (.42-.64)
Cakes/pastries	0.30 (.12-.46)	0.52 (.40-.62)	0.40 (.25-.53)	0.61 (.50-.70)
Cookies	0.51 (.36-.63)	0.34 (.20-.47)	0.29 (.13-.44)	0.45 (.32-.56)
Sweets[5]	0.50 (.35-.63)	0.45 (.32-.56)	0.48 (.34-.60)	0.52 (.40-.62)
Ice cream	0.50 (.35-.63)	0.54 (.42-.64)	0.55 (.42-.66)	0.66 (.56-.74)
Pudding/rice pudding	0.34 (.17-.49)	0.50 (.38-.61)	0.41 (.26-.54)	0.61 (.50-.70)
Pancakes	0.35 (.18-.50)	0.58 (.47-.67)	0.47 (.33-.59)	0.60 (.49-.69)
Sweet spreads	0.61 (.48-.71)	0.73 (.65-.79)	0.65 (.54-.74)	0.70 (.61-.77)
Hazelnut spread	0.53 (.38-.65)	0.66 (.56-.74)	0.62 (.50-.71)	0.77 (.70-.83)
Salty snacks	0.47 (.31-.60)	0.66 (.56-.74)	0.53 (.40-.64)	0.59 (.48-.68)
Nuts	0.28 (.10-.44)	0.45 (.32-.56)	0.32 (.16-.46)	0.44 (.31-.56)
Mean	0.45	0.56	0.46	0.58

Abbreviation: CI (confidence interval), SES (socio-economic status)

[a]According to Winkler [22]

*Non-overlapping 95% confidence intervals (bold) were considered statistical significant

[1]Brown bread, brown bun

[2]White bread, white bun

[3]Curd, yoghurt, soured milk

[4]Burger, doner kebab, fried/grilled sausage, curried sausage

[5]Sweets, fruit chews, chocolate
